# Cost-utility analysis of benzodiazepine deprescription in primary care: a cohort study

**DOI:** 10.1186/s12913-026-14149-2

**Published:** 2026-02-17

**Authors:** Ingrid Ferrer López, Zuzana Špacírová, Antonio Olry de Labry-Lima, Alicia Gutierrez-Valencia, Miguel Florencio Sayago, Maria Martinez Celdran, Maria Antonia Sumariva Bernal, Clara Bermúdez-Tamayo

**Affiliations:** 1 Primary Care Health District, Clinical Management Unit Pharmacy Primary Care, Andalusian Health Service, Seville, Spain; 2 Andalusian School of Public Health, Granada, Spain; 3 Ciber de Epidemiología y Salud Pública- CIBERESP, Barcelona, Spain; 4 Ibs.Granada. Instituto Biosanitario de Granada, Granada, Spain; 5 University of Granada, Campus de la Cartuja s/n, Granada, 18011 Spain; 6 La Guancha Primary Care Facility, Tenerife Primary Care Management Board, The Canary Islands Health Service. Santa Cruz de Tenerife, Canary Islands, Spain; 7 Primary Care Health District, Clinical Management La Candelaria Primary Care, Andalusian Health Service, Seville, Spain; 8 Virgen Macarena Hospital, Critical Care and Emergency Department, Seville, Spain

**Keywords:** Benzodiazepines, Deprescription, Cost-utility analysis, Primary care, QALYs, Healthcare utilization, Charlson Comorbidity Index, Health economics

## Abstract

**Background:**

Long-term benzodiazepine (BZD) use is associated with adverse clinical outcomes and increased healthcare utilization. Although deprescription programs aim to reduce these risks, the net economic impact of BZD discontinuation—particularly in terms of healthcare resource use—remains unclear. This study aimed to evaluate the cost-utility of BZD deprescription intervention in primary care, assessing its impact on direct healthcare costs, service utilization, and quality-adjusted life years (QALYs).

**Methods:**

We conducted a retrospective cohort study using data from 333 patients enrolled in the BenzoStopJuntos deprescription program in Andalusia, Spain. Participants were classified according to BZD status at six-month follow-up: continuation vs. discontinuation. Healthcare resource use (primary care, hospital specialist consultations, primary care emergency, and hospital emergency visits) was extracted from routine electronic health records and monetized using standardized national tariffs (2024 euros). Health outcomes were estimated using the COOP/WONCA charts, from which utility scores and QALYs were derived. We conducted a cost-utility analysis (CUA) from the public payer perspective and calculated the incremental cost-effectiveness ratio (ICER). Probabilistic sensitivity analysis (PSA) was performed using Monte Carlo simulations. Covariate-adjusted Poisson regression models were used to analyze the association between BZD status and healthcare utilization. Subgroup analyses were conducted by comorbidity level (Charlson Comorbidity Index) and sex.

**Results:**

Of the 333 participants, 45.6% discontinued BZD use at follow-up. The discontinuation group incurred lower mean total healthcare costs (€753.2 vs. €826.6), with a non-significant difference of –€73.4, and higher mean QALYs, resulting in an incremental QALY gain of 0.033. The ICER was –€2,185 per QALY, indicating that deprescription was a dominant strategy. Adjusted Poisson models showed a significant 45% reduction in primary care emergency visits in the discontinuation group (IRR = 0.55, 95% CI: 0.37–0.82; *p* = 0.003), with no significant differences in routine primary care, hospital specialist consultations, or hospital emergency visits. PSA confirmed the robustness of these findings, with the majority of simulations in the southeast quadrant of the cost-effectiveness plane. Subgroup analyses showed consistent cost-effectiveness, with greater dominance observed in participants with low to moderate comorbidity.

**Conclusions:**

BZD deprescription through a structured primary care intervention is a cost-effective and clinically safe strategy. It does not result in increased short-term healthcare utilization and may reduce emergency care demands. These findings support the integration of deprescription protocols into routine clinical practice and the expansion of similar interventions in public health systems.

**Trial Registration:**

ClinicalTrials.gov NCT06209827. 01/12/21

## Introduction

The long-term use of benzodiazepines (BZD), prescribed primarily for anxiety, insomnia, and related psychiatric conditions, presents a significant challenge in modern healthcare systems. Chronic BZD use is associated with cognitive impairment, falls, increased risk of road traffic accidents, dependence, and reduced quality of life [1–3]. In severe cases, long-term use has been linked to elevated suicide risk, while abrupt discontinuation can precipitate serious withdrawal complications such as delirium and seizures [4–6]. Despite these risks, the prevalence of long-term BZD use remains high, driven by entrenched prescribing practices, perceived short-term efficacy, and barriers to discontinuation such as withdrawal symptoms and patient resistance [7]. Addressing this issue requires targeted interventions to mitigate health risks, enhance patient outcomes, and potentially improve QoL while reducing healthcare costs [8].

Deprescribing, a systematic process of tapering or discontinuing medications when their risks outweigh their benefits, has emerged as a promising strategy for addressing the overuse of BZDs [9]. Evidence suggests that deprescribing can improve health outcomes and lower healthcare costs by reducing medication-related adverse effects [10]. Discontinuing BZDs may enhance QoL by alleviating cognitive impairment and physical dependence. Conversely, withdrawal symptoms, unaddressed underlying conditions such as anxiety or insomnia, and the psychological distress of deprescribing may negatively affect QoL, at least in the short term [11, 12]. This duality highlights the need for a nuanced understanding of deprescribing outcomes.

BZD deprescription is strongly supported by clinical practice guidelines and evidence. The Canadian Deprescribing Network developed evidence-based guidelines, recommending deprescribing for older adults (strong recommendation) and adults aged 18–64 using BZDs for more than 4 weeks (conditional recommendation) [8]. The UK National Institute for Health and Care Excellence (NICE) provides comprehensive guidance on benzodiazepine withdrawal [13]. Multiple systematic reviews confirm feasibility and safety; a 2023 meta-analysis reported discontinuation rates of 27–80%, with most studies showing no differences in withdrawal symptoms or sleep quality between discontinuation and continuation groups [14]. A 2018 Cochrane review [15] and 2021 Family Practice systematic review support guided discontinuation programs [2]. Regarding quality of life, while withdrawal symptoms may occur during tapering, these are usually mild and transient when conducted gradually. Studies report quality of life decline in those continuing benzodiazepines versus those discontinuing over medium-term follow-up, indicating long-term benefits outweigh temporary withdrawal effects [16, 17].

The economic implications of deprescribing add another layer of complexity. While reducing long-term costs through decreased emergency department visits, hospitalizations, and other interventions for adverse drug events [18], deprescribing may increase short-term healthcare resource utilization. Patients undergoing deprescription might require more frequent primary care visits, mental health support, or interventions to manage withdrawal symptoms [19, 20]. These short-term demands could offset anticipated cost savings, particularly for vulnerable populations such as individuals with complex comorbidities.

A gender-based perspective provides further insight into the patterns of BZD use and deprescribing interventions. Women in Spain exhibit significantly higher rates of BZD consumption than men, with long-term use reported in 9.7% of women versus 4% of men [21, 22]. Such disparities, influenced by biological and sociocultural factors, affect access to care and utilization patterns, supporting the need for gender-sensitive analyses. In addition, comorbidities also significantly influence healthcare resource utilization. Higher scores on the Charlson Comorbidity Index (CCI) are associated with increased healthcare costs, driven by more frequent primary care visits, hospitalizations, and hospital specialist consultations [23]. For BZD users, the interplay of polypharmacy, adverse drug effects, and comorbid conditions amplifies healthcare resource use. While deprescribing in such high-risk populations may alleviate some long-term burdens, it may also increase short-term resource utilization, particularly among individuals with higher CCI scores.

In Spain—where BZD consumption ranks among the highest globally—these considerations are especially relevant. Spain’s elevated BZD consumption reflects both regulatory and cultural factors. While BZDs require medical prescription, the system permits repeat prescribing without mandatory follow-up, and enforcement of prescribing guidelines has been inconsistent [24]. Cultural normalization of BZD use for common complaints and the ability of patients to change providers within the public system further contribute to chronic use patterns [25]. In Andalusia, elevated use is partly driven by an aging population and entrenched prescribing practices. Programs such as *BenzoStopJuntos* [9, 26] have shown promise in reducing inappropriate prescribing. While pharmacological savings are an expected outcome of deprescription, they represent only one dimension of its broader impact [27, 28]. The true challenge lies in understanding how deprescription affects downstream healthcare use, clinical outcomes, and quality of life. Although it may reduce adverse events, polypharmacy, and emergency visits, it could also temporarily increase demands on primary care, mental health services, or patient support programs. Moreover, quality-of-life gains are not guaranteed, particularly in individuals with chronic use or comorbidities.

A key question—still not fully answered by existing evidence—is whether short-term savings from deprescription are offset by increased healthcare utilization during the transition period. Patients may require additional follow-up or support to manage underlying conditions such as anxiety or insomnia. To address this, we evaluated real-world patterns of healthcare use, direct medical costs, and quality-adjusted life years (QALYs) in primary care. This study provides a comprehensive cost-utility assessment of BZD deprescription, comparing those who discontinued use with those who continued. The findings aim to clarify whether the economic value of deprescription truly extends beyond medication savings or merely shifts costs within the healthcare system.

## Methods

### Study design and context

This study employed a quasi-experimental, pre-post cohort design to evaluate the economic and clinical impact of BZD deprescription. The analysis was conducted in a real-world primary care setting within the framework of the “BenzoStopJuntos” program in Andalusia, Spain. A cost-utility approach was applied to compare healthcare resource utilization, direct medical costs, and QALYs between patients who successfully discontinued BZDs and those who continued use over a 6-month follow-up period.

The BenzoStopJuntos program is a multicomponent intervention implemented in primary care that includes: (1) educational materials adapted from the Canadian Medication Appropriateness and Deprescribing Network, translated into Spanish; (2) structured cognitive-behavioral support provided by trained primary care professionals; (3) personalized gradual BZD reduction tailored to baseline dose, duration of use, and primary indication, typically following 25% reductions every 2–4 weeks; (4) scheduled follow-ups at 2, 4, and 6-month intervals; and (5) coordination among family physicians, nurses, and primary care pharmacists. Transfer to long-acting benzodiazepines was not systematic but remained at individual clinical discretion. Non-pharmacological strategies were first-line interventions, including sleep hygiene and relaxation techniques for insomnia, and cognitive-behavioral techniques for anxiety. When clinically necessary, alternative medications (e.g., melatonin, SSRIs) were considered. The program emphasized non-pharmacological approaches and discouraged inappropriate substitution with other psychotropic medications. Program implementation costs were not separately analyzed as the intervention utilized existing primary care resources and publicly available educational materials.

### Perspective and time horizon

The economic evaluation was conducted from the perspective of the Spanish public healthcare system, including only direct medical costs borne by healthcare providers. A 6-month time horizon was selected to reflect short-term changes in healthcare resource utilization and health outcomes resulting from the deprescription process. As the follow-up period was limited to six months, no discounting was applied to costs or effects.

### Study population

The study population included adults aged 18 years or older who were consumption BZD for more than 4 weeks and enrolled in the “*BenzoStopJuntos*” deprescription program and had used BZDs for more than four weeks. Participants with serious mental illness, terminal conditions, alcohol dependence, dementia, or intellectual disability were excluded. Individuals were categorized into two groups based on their BZD use status at the end of follow-up: those who had successfully discontinued use (BZD Discontinuation) and those who continued treatment (BZD Continuation). Successful deprescription was defined as no dispensing of BZDs during the final two consecutive months (months 5 and 6) of the six-month follow-up period. This two-month criterion was selected to distinguish sustained discontinuation from temporary interruptions in dispensing (e.g., due to medication stockpiling) and to ensure patients had completed the typical acute withdrawal period, which resolves within 2–4 weeks with gradual tapering. This approach is consistent with definitions used in prior BZD deprescription studies.

### Comparators

The analysis compared two groups of participants enrolled in the BenzoStopJuntos program: those who successfully discontinued BZD use during follow-up (“BZD Discontinuation” group) and those who continued treatment (“BZD Continuation” group). Both groups received the same structured intervention, including educational materials, cognitive-behavioral support, and scheduled follow-ups. Group assignment was observational and based on participants’ BZD dispensing status at the end of the 6-month follow-up period after intervention initiation. All participants were followed for six months from enrollment in the BenzoStopJuntos program (baseline), regardless of their discontinuation status.

### Outcomes and measures

The primary outcomes included healthcare resource utilization (number of visits to primary care, hospital specialist consultations, and emergency services), direct medical costs, and health-related quality of life. Quality of life was assessed using the WONCA-COOP functional health status chart, a 7-item instrument evaluating physical fitness, emotional well-being, daily activities, social functioning, pain, perceived health change, and general health. Each item was scored from 1 (best) to 5 (worst), resulting in a total score range of 7 to 35. [29, 30]. The COOP/WONCA charts are a validated generic measure of functional health status in primary care but not a preference-based multi-attribute utility instrument (e.g., EQ-5D, SF-6D), and no validated preference-based tariff exists for converting COOP/WONCA scores to utility values.

Given that data were collected retrospectively from routine clinical practice and preference-based instruments (e.g., EQ-5D, SF-6D) were not available, we derived health state utility values from COOP/WONCA scores using a linear transformation: utility = (35 – WONCA score)/28. This transformation scales the instrument’s total score range (7–35) to a 0–1 utility scale, where 0 represents the worst health state and 1 represents perfect health.

The COOP/WONCA instrument assesses health domains (physical functioning, emotional well-being, pain, and social activities) that are conceptually aligned with the health state descriptors used in preference-based instruments. The linear transformation assumes a monotonic relationship between functional health status and health-related utility, preserving relative differences between groups. This approach has been applied in similar contexts when validated preference-based measures are unavailable in retrospective data [31, 32]. Importantly, both study groups underwent identical assessments using the same methodology, ensuring that any potential systematic measurement characteristics affect both groups equally and do not bias comparative estimates. QALYs were calculated assuming linear utility transition between baseline and 6-month assessments: QALY = [(utility_baseline + utility_6months)/2] × 0.5.

### Data collection

Data collection was conducted by trained clinical staff following a standardized protocol. Data on BZD use, quality of life, and healthcare visits were collected at baseline and after 6 months. Clinical and dispensing data were retrospectively retrieved from the Andalusian Health Service electronic health records by authorized personnel. Data were anonymized prior to analysis. Quality control procedures included random checks and cross-validation of data entries to ensure reliability and completeness.

### Health care resources use and costs

Unit costs were obtained from the official tariffs published in the Official Gazette of the Autonomous Community of Andalusia [33, 34] and reflect the standard public reimbursement rates applicable to each service category. Specifically, the following unit costs (in 2024 euros) were used: €64.07 for a primary care visit, €81.38 for a hospital specialist consultation, €73.30 for a primary care emergency visit, and €215.06 for a hospital emergency visit. All costs were expressed in 2024 euros. Historical unit costs were updated to 2024 values using the Consumer Price Index (CPI) published by the Spanish National Institute of Statistics [35]. No currency conversions were required. Cost estimates were calculated at the individual level by multiplying the number of healthcare events by their respective unit costs and summing them across categories.

Pharmaceutical costs, diagnostic procedures, and indirect costs (such as productivity loss or informal care) were not included in the analysis. This exclusion was based on methodological, operational, and conceptual grounds, consistent with the objective of assessing the broader healthcare consequences of BZD deprescription from a public healthcare system perspective. First, pharmaceutical costs were deliberately excluded because the analysis does not aim to quantify the predictable savings derived from discontinuing a low-cost drug, but rather to examine what happens after deprescription. We assumed that individuals who stop using BZD no longer generate pharmaceutical expenditure for these drugs, making this a baseline assumption rather than a variable outcome. Second, diagnostic procedures (e.g., laboratory or imaging tests) were excluded due to their inconsistent availability in the health records used and the expected low frequency of such services during the six-month follow-up. Estimating these costs would have required assumptions unsupported by reliable data, risking biased estimates. Finally, indirect costs were not included as the analysis was conducted from the payer’s perspective. Given the participants’ average age, most were likely retired, rendering productivity loss largely irrelevant.

### Statistical analysis

Descriptive statistics were calculated to summarize healthcare resource utilization, total costs, and baseline demographic and clinical characteristics of the study population. Means, standard errors, and proportions were reported as appropriate.

#### Cost-consequence analysis (CCA)

A cost-consequence analysis was conducted to compare direct healthcare costs and resource utilization between participants who continued using benzodiazepines and those who successfully discontinued use during the 6-month follow-up. The analysis focused on four categories of healthcare resources: primary care visits, hospital specialist consultations, primary care emergency visits, and hospital emergency visits.

Total healthcare costs per participant were calculated as the sum of costs across all resource categories. Differences in mean costs between groups were reported alongside adjusted estimates. To examine the factors associated with healthcare resource use, Poisson regression models were applied separately for each resource category. These models included covariates such as age, sex, Charlson Comorbidity Index (CCI), duration of BZD use, annual income, and the presence of minor mental disorders. Poisson regression was selected to account for the count nature and potential overdispersion of healthcare utilization data, and to allow for the estimation of incidence rate ratios (IRRs) reflecting the influence of each covariate on the frequency of resource use.

#### Cost-utility analysis (CUA)

To assess the cost-effectiveness of BZD deprescription, a cost-utility analysis was performed using Quality-Adjusted Life Years (QALYs) as the outcome measure. The Incremental Cost-Effectiveness Ratio (ICER) was calculated by dividing the difference in mean costs between groups by the difference in mean QALYs.

To assess the robustness of the cost-effectiveness results, a Probabilistic Sensitivity Analysis (PSA) was performed with 1000 Monte Carlo simulations. Costs were modeled using gamma distributions with shape (α) and scale (β) parameters derived from observed data: α = μ^2^/σ^2^ and β = σ^2^/μ, where μ is the mean and σ^2^ is the variance. QALYs were modeled using beta distributions bounded between 0 and 1, with parameters estimated from observed utility score means and standard deviations. Each iteration generated a pair of simulated cost and QALY values used to estimate the distribution of ICERs.

Results were visualized using a cost-effectiveness plane and an ICER distribution histogram with a superimposed density curve. All statistical analyses were conducted using Stata version 14.

### Ethical considerations

This study was conducted in compliance with the principles of the Declaration of Helsinki and applicable national regulations on biomedical research involving human subjects. Ethical approval was granted by the Andalusian Health Research Ethics Committee (Ref. IFLBZD16). All participants provided written informed consent prior to enrollment. The consent process included detailed information about the study.

## Results

### Participants characteristics

The study included 333 participants with a mean age of 66.7 years; 70% were women. Most had annual incomes ≤€18,000 (70.6%) and low to moderate comorbidity, with 41.5% scoring CCI low 0–2 and 38.8% scoring CCI moderate 3–4. The main indications for BZD use were insomnia (57%) and anxiety (24%). The average duration of BZD use was 4 years, and baseline health-related quality of life was 0.59 on a 0–1 scale (Table 1).

**Table 1:** Baseline characteristics

Variables		N (%)
Sex	Men	100 (30.03)
	Women	233 (69.97)
Being treated by mental minor disorder	No	321 (96.41)
	Yes	12 (3.60)
Income (€/year)	≤ 18,000	235 (70.57)
	> 18,000	98 (29.43)
Comorbidity Charlson index	No or mild comorbidity (0–2) Moderate comorbidity (3–4) Severe comorbidity (≥5)	137 (41.52) 128 (38.79) 65 (19.70)
Indication for use	Anxiety Insomnia Anxiety and insomnia Others	79 (23.72) 189 (56.76) 29 (8.71) 14 (4.20)
		**Average (SE), range**
Age		66.72 (0.68), 32–95
Duration of BZD use (years)		4.04 (0.28), 0.1–40
Quality of life		0.59 (0.01), 0.035–1

### Healthcare resource use and costs over a 6-month time horizon

After six months, participants who discontinued BZD use had slightly lower total healthcare costs than those who continued (€753 vs. €827), although this difference was not statistically significant. Resource use was generally similar between groups, with the exception of primary care emergency visits, which were significantly less frequent among those who discontinued BZDs (0.31 vs. 0.58; *p* = 0.048) (Table 2). These findings remained consistent after multiple imputation. Stratified analyses by sex also showed cost reductions in both men and women who discontinued BZD use, though without statistical significance (Table 3).

**Table 2:** Resources use and total costs after 6-month follow-up

	Complete case						
	Use/person		Unit cost (€)	Cost/person		Difference mean cost per person	p-value^*^
	**BZD Continuation N = 223** (67.6%) (mean)	**BZD Discontinuation N = 107** (32.4%) (mean)		Yes BZD **N = 223** (67.6%) (mean)	No BZD **N = 107** (32.4%) (mean)		
Primary care visits	9.03	8.20	64.07	579	525	−54	0.582
Hospital specialist consultations	1.67	1.63	81.38	136	133	−3	0.921
Primary care emergency visits	0.58	0.31	73.3	42	23	−20	**0.048**
Hospital emergency visits	0.32	0.34	215.06	69	72	2.92	0.915
Total costs				827	753	−73	0.500

^*^Mann-Whitney test. Unit costs updated to 2024 prices

**Table 3:** Comparison of healthcare resource utilization and costs between benzodiazepine deprescription and continuation groups, stratified by sex

Variable	BZD Continuation (N = 223)	BZD Discontinuation (N = 107)	p-value*
Men; N(%)	64 (64%)	36 (36%)	
Primary care visits; mean	8.73	8.61	
Hospital Specialist consultations; mean	1.97	2.11	
Primary care emergency visits; mean	0.70	0.36	
Hospital emergency visits; mean	0.36	0.36	
Total costs; mean	849€	828€	0.613
Women; N(%)	159 (69%)	71 (31%)	
Primary care visits; mean	9.15	7.99	
Hospital Specialist consultations; mean	1.55	1.39	
Primary care emergency visits; mean	0.53	0.28	
Hospital emergency visits; mean	0.31	0.32	
Total costs; mean	818€	715€	0.2169

^*^Mann-Whitney test

In adjusted regression models, BZD discontinuation was associated with a modest, non-significant reduction in primary care visits (*p* = 0.066). Visit rates increased with age (IRR = 1.01; *p* < 0.001) and comorbidity level (IRR = 1.18 for moderate, 1.31 for high; both *p* < 0.01), while higher income was associated with fewer visits (IRR = 0.83; *p* < 0.001). Individuals with minor mental disorders had substantially more visits (IRR = 1.76; *p* < 0.001) (Table 4).

**Table 4:** Incidence rate ratios for predictors of healthcare resource utilization across different models and cost-effectiveness analysis

Model	Predictor	IRR	p-value	95% CI
Primary care visits				
	**BZD Continuation**	0.93	0.066	0.84, 1.01
	Age	1.01	< 0.001	1.01, 1.02
	CCI 1: No or mild comorbidity (0–2)	1.18	0.006	1.05, 1.28
	CCI 2 Moderate comorbidity (3–4)	1.31	< 0.001	1.13, 1.49
	Income	0.83	< 0.001	0.72, 0.91
	Minor Mental Disorder	1.76	< 0.001	1.46, 2.09
Hospital Specialist consultations				
	**BZD Continuation**	1.04	0.708	0.86, 1.21
	Age	0.98	0.002	0.97, 0.99
	CCI 1: No or mild comorbidity (0–2)	1.83	< 0.001	1.34, 2.31
	CCI 2 Moderate comorbidity (3–4)	2.94	< 0.001	1.92, 3.94
	Income	0.77	0.010	0.64, 0.94
	Sex (Men)	1.26	0.015	1.04, 1.52
Primary care emergency visits				
	**BZD Continuation**	0.55	0.003	0.44, 0.81
	Age	0.99	0.510	0.98, 1.01
	CCI 1: No or mild comorbidity (0–2)	0.82	0.409	0.50, 1.32
	CCI 2 Moderate comorbidity (3–4)	1.01	0.988	0.55, 1.81
	Income	0.29	< 0.001	0.17, 0.49
	Minor Mental Disorder	1.09	0.840	0.49, 2.40
	Sex (Men)	1.42	0.044	1.01, 1.81
Hospital emergency visits				
	**BZD Continuation**	0.97	0.886	0.63, 1.52
	Age	1.00	0.933	0.98, 1.02
	CCI 1: No or mild comorbidity (0–2)	1.00	0.992	0.53, 1.85
	CCI 2 Moderate comorbidity (3–4)	2.47	0.011	1.21, 3.72
	Income	0.79	0.326	0.50, 1.26
	Minor Mental Disorder	3.57	< 0.001	2.01, 4.32
	Sex (Men)	0.88	0.561	0.56, 1.37
	**BZD Continuation (Mean)**	**BZD Discontinuation (Mean)**	**Difference (No-Yes)**	
Costs (CT) (€)	826.60 (SE: 45.79)	753.20 (SE: 58.43)	−73.40	
QALYs	0.0275 (SE: 0.0087)	0.0611 (SE: 0.0092)	+0.0336	
Incremental Cost-Effectiveness Ratio (ICER)	-	-	-€2,185.71 per QALY	

*BZD, Benzodiazepine; CCI, Charlson Comorbidity Index, QALY, Quality-adjusted life-years, IRR:Incidence Rate Ratio*

For hospital specialist consultations care, BZD discontinuation had no effect (*p* = 0.708), but male sex (IRR = 1.26; *p* = 0.015), lower age (IRR = 0.98; *p* = 0.002), lower income (IRR = 0.77; *p* = 0.010), and higher comorbidity (IRR = 1.83 for moderate, 2.94 for high; both *p* < 0.001) were all associated with increased utilization.

Emergency visits in primary care were significantly reduced among participants who discontinued BZD use (IRR = 0.55; *p* = 0.003). Male sex (IRR = 1.42; *p* = 0.044) and lower income (IRR = 0.29; *p* < 0.001) were also associated with higher emergency visit rates.

Hospital emergency visits did not differ by BZD status (*p* = 0.886), but were significantly higher among participants with severe comorbidity (IRR = 2.47; *p* = 0.011) and those with minor mental disorders (IRR = 3.57; *p* < 0.001).

### Cost-utility

The cost-utility analysis showed that BZD discontinuation was both less costly and more effective than continued use. The mean healthcare cost per participant was €753 in the discontinuation group and €827 in the continuation group. Average QALYs were 0.0611 for those who discontinued and 0.0275 for those who continued, yielding an incremental gain of 0.0336 QALYs. This resulted in a negative ICER of –€2,185.71 per QALY, indicating that discontinuation was the dominant strategy (Table 4).

**Fig. 1:**
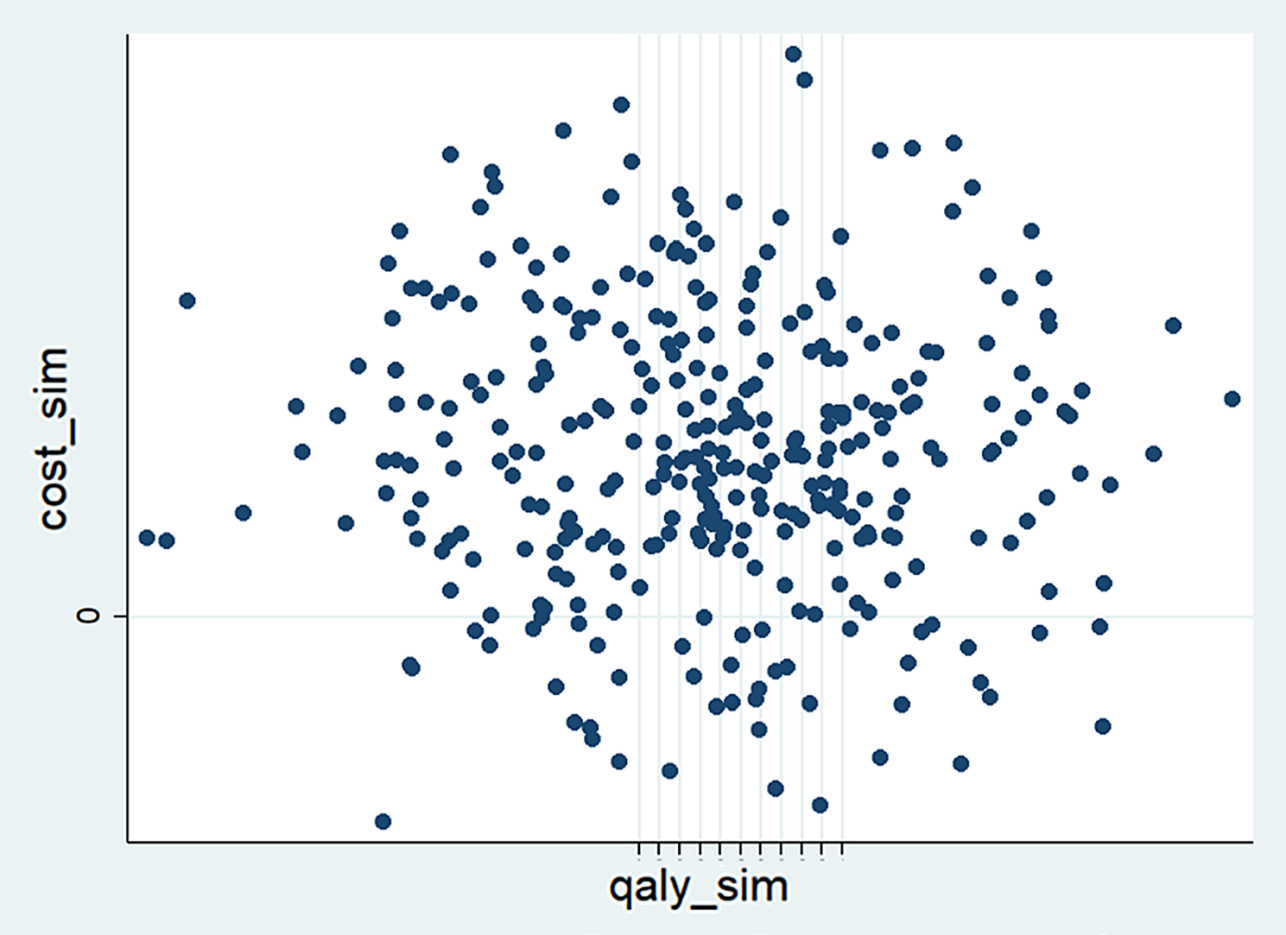
Probabilistic sensitivity analysis (PSA) comparing the BZD discontinuation group with the BZD Continuation

The probabilistic sensitivity analysis (PSA), based on 1000 simulations, confirmed the robustness of these findings. Most simulations clustered in the lower-right quadrant of the cost-effectiveness plane, supporting the dominance of BZD discontinuation—that is, it resulted in both lower costs and greater health benefits. While a small number of simulations fell in the upper-right quadrant, suggesting increased costs for improved outcomes, very few indicated reduced effectiveness. The ICER distribution was centered near zero, reinforcing the consistency of the dominant cost-effectiveness profile (Fig. 1).

### Subgroup analysis

**Fig. 2:**
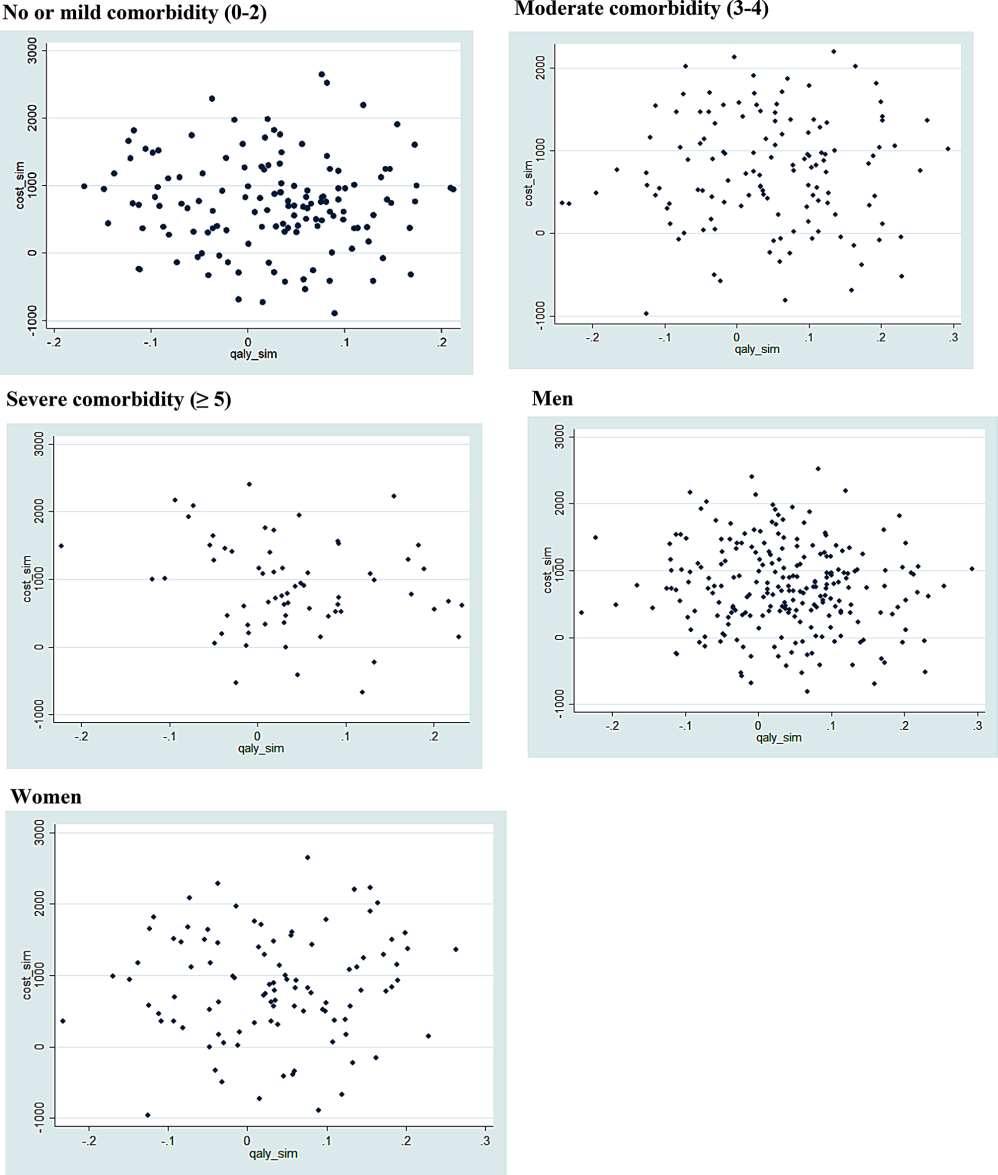
Probabilistic sensitivity analysis (PSA) stratified by comorbidity and sex

Cost-effectiveness planes stratified by comorbidity level demonstrated that BZD discontinuation remained a dominant or cost-effective strategy across all clinical subgroups. In participants with no or mild comorbidity, the majority of simulations clustered in the lower-right quadrant, indicating scenarios where discontinuation was both less costly and more effective. A smaller proportion of simulations fell in the upper-right quadrant, suggesting improved outcomes at a higher cost, while points in the left quadrants were minimal, reflecting few cases of reduced effectiveness (Fig. 2).

In the moderate comorbidity group, the distribution was more dispersed, though the overall pattern remained favorable. Most simulations supported the dominance or cost-effectiveness of discontinuation, while a larger share of points in the upper-right quadrant reflected increased variability in cost-outcome trade-offs. In the severe comorbidity subgroup, a similar pattern emerged: although many simulations showed cost savings with health gains, a substantial number indicated higher costs associated with improved outcomes. These findings underscore the potential efficiency of deprescribing even in more complex patients, contingent on the cost-effectiveness threshold applied.

Sex-stratified analyses showed consistent trends. In both male and female subgroups, most simulations favored BZD discontinuation, with a concentration of points in the dominant quadrant. The female subgroup exhibited greater dispersion, potentially reflecting heterogeneity in baseline health status or healthcare use. Nevertheless, scenarios falling into the left quadrants—representing reduced effectiveness—were uncommon in either sex, reinforcing the robustness of the intervention across demographic strata (Fig. 2).

## Discussion

This study enhances the evidence base on the impact of BZD deprescription in primary care. While direct medication savings are a well-recognized advantage of BZD discontinuation, there is ongoing debate about whether these savings are offset by increased healthcare resource use elsewhere in the system [36].

Our results indicate that BZD deprescription led to either stable or reduced healthcare resource utilization in the short term, most notably a significant reduction in primary care emergency visits, and without a compensatory increase in other domains such as scheduled visits or hospital specialist consultations.

While Burke et al. [37] reported a sharp reduction in overall outpatient visits following deprescription (from 25.4 to 4.4 annually), they did not differentiate between routine and emergency care, limiting interpretability. Similarly, Trépel et al. [38] included general practitioner contacts in their cost-effectiveness model but did not report service frequency or types of visits. Kollen et al. [39] reported that a national policy restricting BZD reimbursement resulted in decreased prescription days over a two-year period without evidence of increased healthcare visits. Similarly, Burke et al. [40] observed a pronounced decline in outpatient visits after BZD detoxification among long-term users.

Participants who discontinued BZDs showed a slight, non-significant reduction in primary care visits. This aligns with prior studies [41, 42] and Reeve et al., which found that outpatient service use generally remains stable after BZD withdrawal, and is more strongly influenced by underlying morbidity and mental health than by BZD status itself. Our adjusted models confirm that age, comorbidity, income, and the presence of minor mental disorders were key drivers of primary care demand [43].

Our cost-utility analysis identified deprescription as a dominant strategy—less costly and more effective. This result was robust across probabilistic sensitivity analyses and consistent in subgroup analyses by sex and comorbidity. It yielded greater benefits in patients with low to moderate comorbidity, supporting previous findings from the OPERAM study [44]. In high-comorbidity subgroups, outcomes were more variable, a result consistent with prior literature showing mixed effects in more complex populations [43]. These findings also compare favorably to previous deprescription programs. like D-PRESCRIBE [45] and the trial by Vicens et al. [11].

This study has several limitations that must be considered when interpreting the results. The six-month follow-up period may not fully capture the long-term effects of BZD deprescription, particularly regarding changes in healthcare resource use and quality of life. Second, despite adjustments in the statistical models, unmeasured variables, such as social support, psychological characteristics of patients, or undocumented interventions, may have influenced the observed results. The cost analysis focused solely on direct healthcare costs, excluding diagnostic tests (laboratory analyses, imaging studies), pharmaceutical expenses (including the cost of benzodiazepines themselves and potential alternative treatments). Participants who enrolled in this study likely represent a more motivated subset of BZD users willing to attempt deprescription. Patient attachment to benzodiazepines and resistance to withdrawal are well-documented barriers to deprescription in clinical practice. Our findings may not fully generalize to patients who are unwilling to consider discontinuation or who have strong beliefs about medication necessity. Real-world implementation of deprescription programs may require additional engagement strategies, including motivational interviewing and tailored communication, to reach patients beyond those already motivated to change.

Our utility estimation relied on a linear transformation of COOP/WONCA functional health scores rather than a validated preference-based tariff, as retrospective data collection precluded use of instruments such as EQ-5D. While this introduces measurement uncertainty, several factors support the validity of our findings. The transformation was applied identically to both groups, ensuring unbiased comparative estimates. Our probabilistic sensitivity analysis demonstrated that cost-effectiveness conclusions remained robust across the uncertainty range in both costs and utilities. Furthermore, the finding of BZD discontinuation as a dominant strategy (simultaneously cost-saving and more effective) is less sensitive to precise utility quantification than borderline cost-effectiveness ratios would be. Calculation assumed linear utility transition between time points rather than capturing the precise timing of quality-of-life changes during deprescription. However, this method was applied identically to both groups, ensuring unbiased comparative estimates.

Our results align with studies emphasizing the short-term cost-effectiveness of continued BZD use in specific populations. For instance, Orly de Labry Lima et al. [46] reported cost savings in primary care settings when BZDs were continued under strict supervision, emphasizing the importance of patient-specific approaches [46]. Similarly, McCarthy et al. [47] highlighted that patients who supported continued BZD use reported improved health-related quality of life, despite the potential for long-term dependency.

In contrast, the central premise of deprescription is supported by studies that link it to significant reductions in polypharmacy-related adverse events. Barnett and Garfinkel [48] stressed that deprescription of BZDs could mitigate risks such as falls and cognitive decline, particularly in older adults. Our subgroup analyses further corroborate these insights. For example, patients with severe comorbidities often benefited economically and health-wise from interventions aligned with deprescription strategies, echoing findings by Kornholt et al. that suggest targeted deprescription in high-risk populations yields optimal outcomes [49].

Overall, while our analysis demonstrates that continued BZD use can be cost-effective and beneficial in certain populations, the broader literature and our subgroup findings advocate for deprescription as a preferred strategy in patients with lower risks of withdrawal and higher susceptibility to BZD-related adverse events. Future research should focus on long-term outcomes and integrate patient-centered approaches to refine deprescription protocols. These findings provide valuable insights for healthcare policymakers aiming to optimize deprescribing strategies and reduce inappropriate medication use while balancing clinical and economic outcomes.

Benzodiazepine deprescription implemented in primary care was cost-effective, clinically safe, and does not increase short-term healthcare utilization. Our findings support the integration of deprescription into routine clinical practice and offer practical implications for improving prescribing quality in aging populations.

## Data Availability

The datasets used and/or analyzed during the current study are available from the corresponding author on reasonable request.

## Abbreviations

BZD: Benzodiazepine
CCA: Cost-Consequence Analysis
CCI: Charlson Comorbidity Index
CPI: Consumer Price Index
CUA: Cost-Utility Analysis
ICER: Incremental Cost-Effectiveness Ratio
IRR: Incidence Rate Ratio
PSA: Probabilistic Sensitivity Analysis
QALY: Quality-Adjusted Life Year
QoL: Quality of Life
SSRI: Selective Serotonin Reuptake Inhibitor
